# STAT3 Activation in Glioblastoma: Biochemical and Therapeutic Implications

**DOI:** 10.3390/cancers6010376

**Published:** 2014-02-10

**Authors:** Jennifer E. Kim, Mira Patel, Jacob Ruzevick, Christopher M. Jackson, Michael Lim

**Affiliations:** Department of Neurosurgery, The Johns Hopkins University School of Medicine, 600 N. Wolfe St., Phipps Building Rm 123, Baltimore, MD 21287, USA; E-Mails: jkim256@jhmi.edu (J.E.K.); mpatel50@jhmi.edu (M.P.); jruzevick@jhmi.edu (J.R.); cjacks53@jhmi.edu (C.M.J.)

**Keywords:** glioblastoma, STAT3, immunotherapy

## Abstract

Signal transducer and activator of transcription 3 (STAT3) is a potent regulator of gliomagenesis through its induction of angiogenesis, host immunosuppression, and tumor invasion. Gain of function mutations result in constitutive activation of STAT3 in glioma cells, making STAT3 an attractive target for inhibition in cancer therapy. Nevertheless, some studies show that STAT3 also participates in terminal differentiation and apoptosis of various cell lines and in glioma with phosphatase and tensin homolog (PTEN)-deficient genetic backgrounds. In light of these findings, the utility of STAT3 as a prognostic indicator and as a target of drug therapies will be contingent on a more nuanced understanding of its pro- and anti-tumorigenic effects.

## 1. Introduction

Glioblastoma multiforme (GBM) is the most common primary malignancy of the central nervous system (CNS), with an incidence of 3.19 per 100,000 and a five-year survival rate of less than 5% [1,2]. GBM’s molecular heterogeneity, genomic instability, radio- and chemo-resistance, infiltrative capacity, and immune evasion are important contributing factors in GBM pathogenesis. Though the mechanisms of treatment resistance and recurrence are not yet fully understood and remain a limiting factor for conventional therapeutics, significant progress has been made in the last decade toward identifying common motifs in gliomagenesis. Genetic and molecular alterations in the epidermal growth factor receptor (EGFR) [3,4,5,6], molecular target of rapamycin (mTOR) [7,8], and vascular endothelial growth factor (VEGF) [9,10,11] signaling pathways, as well as mutations of isocitrate dehydrogenase (IDH)-1 and -2 [12], PTEN [13] and DNA repair enzyme O6-methylguanine-DNA methyltransferase (MGMT) [14] have been described as possible bases of molecular therapies for both primary and secondary GBMs.

Another potential target of GBM molecular therapy is signal transducer and activator of transcription (STAT) 3, a member of the STAT family of proteins that mediates cytokine signaling and nuclear transcription. STAT3 is an attractive focus for therapeutic intervention since it represents a point of convergence for multiple oncogenic signaling cascades. STAT3 has emerged as a key initiator and master regulator of mesenchymal transformation in malignant gliomas [15]. Constitutive STAT3 activation has been implicated in the suppression of host antitumor immune response, thereby facilitating unregulated tumor growth [16,17,18,19]. STAT3 can be activated by growth factor receptors including EGFR and platelet-derived growth factor receptors (PDGFR), as well as interleukin-6 receptor (IL-6R/gp130), Janus family kinases (JAK), Abl family kinases, and Src family kinases [16,20,21,22,23]. It has also been shown to be a potent regulator of gliomagenesis by inducing local angiogenesis and promoting immune evasion and tumor invasion [17,24].

Several preclinical studies have demonstrated the anti-tumor effects of STAT3 knockdown using small interfering RNA (siRNA), micro-RNA (miRNA), or small molecule inhibitors [17,25,26,27,28]. However, the varying efficacy of these treatments with respect to cell type, *in vitro versus in vivo* model, and therapeutic modality underscore our incomplete understanding of the role of STAT3 activation in GBM. In fact, some studies have suggested that STAT3 can act, paradoxically, as a tumor suppressor by participating in terminal differentiation and apoptosis. The utility of STAT3 as a prognostic indicator and therapeutic target is, therefore, contingent on further clarification of its pro- and anti-tumorigenic effects. Here, we review the role of STAT3 activation in gliomagenesis, and summarize the most recent laboratory and translational strategies for targeted STAT3 inhibition. 

## 2. STAT3 Signaling Pathways

STAT proteins are a family of cytoplasmic transcription factors that are activated by tyrosine kinases and mediate cellular response to inflammatory and proliferative signals [29,30]. These tyrosine kinases include growth factor receptors, such as EGFR and PDGFR, and cytoplasmic enzymes, specifically the JAK and Src kinase families [23]. Phosphorylated STAT proteins dimerize via reciprocal phosphotyrosine-SH2 interactions and undergo nuclear translocation. There, they bind consensus STAT binding proteins, or DNA-response elements of the targeted sequences to regulate transcription and gene expression. Transcription activity can be maximized by serine phosphorylation of STAT dimers by intranuclear protein serine kinases (PSKs) [23,31,32,33]. STAT3 signaling is tightly regulated by several upstream and downstream checkpoints to ensure an appropriate growth response to activation. Inhibitory molecules, such as protein tyrosine phosphatases, act to dephosphorylate and inactivate ligand-receptor complexes and phosphorylated-STAT (pSTAT) dimers [34,35]. Suppressor of cytokine signaling (SOCS) proteins negatively feedback on the JAK/STAT signaling pathway by disrupting or degrading JAKs [36]. In response to cytokine stimulation, protein inhibitor of activated STAT3 (PIAS3) can block STAT protein’s DNA-binding activity, thereby inhibiting gene transcription [37]. Furthermore, STAT3 interacting protein (StIP1) may block STAT3 activation, translocation, and reporter gene induction via overexpression of its STAT3-binding domain [38].

These molecules play a significant role in regulating the STAT3 signaling cascade, and under normal physiologic conditions, represent important checkpoints in the activation and deactivation of cell proliferation [39]. However, under pathologic or experimental conditions, these regulatory molecules serve as natural targets for STAT3 signal disruption or even constitutive activation. Examples of disruptive mediators and their respective targets are listed in Table 1.

**Table 1:** Sites of potential STAT3 signal disruption.

Site of disruption	Potential mediators of disruption
Cell surface receptor-ligand interaction	Ligand/receptor antagonists, *i.e.*, anti-EGF-R antibodies [5,40]
Tyrosine or serine kinase activity	TRK, EGF-R, FBGF-R, JAK, Src, PSK inhibitors [15,20,21,28]
Endogenous STAT3 activity or function	Biological protein inhibitors of STAT3 activity, *i.e.*, SOCS, PIAS3, StIP1 modulators [36,37,41,42]
De-phosphorylation of phospho-STATs	Protein tyrosine or serine phosphatases [25,43]
STAT3 dimerization	Small molecule inhibitors of dimerization [43]
STAT3 nuclear translocation	Small molecule inhibitors of dimerization, inhibitors of nuclear endocytosis [43,44]
STAT3 transcription activation	Antisense or STAT3 decoy oligonucleotide sequences, dominant negative mutants [45,46,47]

## 3. Constitutive Activation

**Figure 1:**
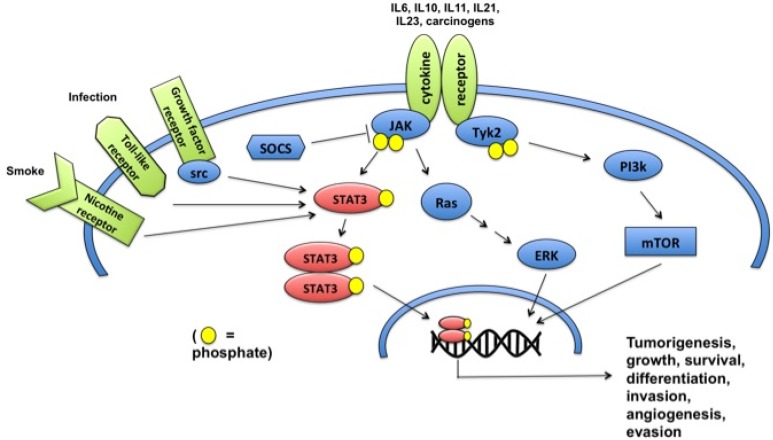
STAT3 as convergence point in the oncogenic pathway.

Persistent STAT3 activation—secondary to intrinsic hyperactivity, aberrant upstream signaling, or defective negative regulation—can lead to abnormal survival and tumorigenesis [48]. Constitutive STAT3 activation has been reported in 50%–90% of human cancers [48,49]. This prevalence can be attributed to STAT3’s position as the convergence point of several major oncogenic signaling pathways (Figure 1), including EGFR, heregulin-2/neuregulin receptor (Her2/Neu), platelet-PDGFR, IL-6R/gp130, c-Met, Abelson leukemia protein (ABL), and Src tyrosine kinases [16,21,22,29,49,50]. Thus, constitutive STAT3 activation commonly results from gain-of-function mutations or overexpression of upstream growth factor receptors or signaling kinases. Additionally, disruption of normal counter-regulatory mechanisms can also initiate or contribute to tumorigenesis. For example, aberrant methylation silencing of SOCS-3 has been associated with constitutive JAK/STAT activity and higher levels of pSTATs in non-small cell lung cancer [51].

Extracellular cytokine dysregulation can also result in abnormal autocrine or paracrine stimulation of cell surface receptors involved in downstream STAT3 signaling. This mechanism has been described in multiple myeloma, where IL-6 overexpression led to increased JAK/STAT3 activity [52], as well as in squamous cell carcinoma of the head and neck, where elevated levels of transforming growth factor-α (TGF-α) and subsequent STAT3 activation abrogated apoptosis of epithelial cells [53]. 

The presence of STAT3 activation in GBMs varies considerably, with detection rates of most retrospective, immunohistochemistry-based studies ranging from 9%–83% [54]. These discrepant data could be attributed to the varying tumor grades of the brain tissue sampled in each study. In 2006, Mizoguchi *et al.* demonstrated similar rates of STAT3 activation in anaplastic astrocytomas (AA) and GBMs (55.6% and 56.4%, respectively) [55]. Abou-Ghazal reported similar results in 2008, with 50% of AA and 51% of GBM samples staining positively for pSTAT3 [56]. Lo *et al.* expanded on these findings by describing a positive correlation between glioma grade and extent of STAT3 activation. Constitutive activation was detected in 60% of primary high grade/malignant gliomas, secondary to JAK2, EGFR and/or EGFRvIII kinase hyperactivity. In comparison, only 27%, 29%, and 57% of Grade I, II, and III gliomas, respectively, were found to have similar activation [41]. These conclusions were further supported by tissue electrophoresis and western blot assays, which also showed a correlation between histopathological grade and STAT3 phosphorylation [57,58,59]. In contrast, Wang *et al.* reported STAT3 activation in only 9% of AA and 9% of GBM samples, and found no correlation with tumor grade [60]. However differing methods in protein detection is a possible explanation for inconsistent results between studies [54].

The mechanisms of STAT3 activation in GBMs are similar to those found in other cancer cell lines. Rahaman *et al.* observed constitutive activation of STAT3 in 90% of human GBM tumors and GBM cell lines. The majority of STAT3 activity in U251 cells was catalyzed by gp130-associated JAKs secondary to IL-6, a cytokine secreted by GBM cells both *in vivo* and *in vitro* [61]. Several studies have shown IL-6 mRNA expression to be significantly elevated in GBM patient samples as compared to those with lower grade gliomas [62,63,64,65]. Furthermore, IL-6 gene amplification has been associated with a significantly lower overall survival among GBM patients [65].

STAT3 gain-of-function mutations have not been reported, but amplifying mutations of STAT3 activators have been identified in several studies. In particular, missense mutations of EGFR genes may lead to constitutive downstream activation, and have been implicated in *de novo* GBMs in older patients [66,67,68]. Bone marrow X-linked (BMX) nonreceptor tyrosine kinase, which is differentially expressed in GBM stem cells, may contribute to self-renewal and tumorigenic potential via STAT3 activation [69].

Contrary to amplifying mutations, decreased expression of STAT3 repressors can cause constitutive activation of STAT3. Brantley *et al.* demonstrated a negative correlation between transcription inhibitor PIAS3 and phospho-STAT3 expression, with subsequent PIAS3 overexpression leading to decreased STAT3 transcriptional activity and cell proliferation [57]. Protein tyrosine phosphatase receptor delta (PTPRD), a STAT3 phosphatase, has been implicated as a suppressor of neuroectoderm-derived tumors. Focal deletions, missense, and nonsense mutations of the PTPRD gene have been identified in up to 41% of GBM samples [70,71]. Furthermore, Veeriah *et al.* observed a greater frequency of PTPRD expression loss in GBM *versus* lower grade gliomas. Decreased PTPRD expression was also predictive of poorer prognosis in GBM patients [71].

## 4. Role of STAT3 in Tumorigenesis

Once activated, STAT3 drives several pro-oncogenic mechanisms to promote cell proliferation and survival, immune suppression, invasion, and angiogenesis.

### 4.1. Survival

Under normal physiologic conditions, STAT3 activity is tightly regulated via ligand-dependent receptor and non-receptor tyrosine phosphorylation [72]. Coordinated interactions with protein tyrosine phosphatases, direct protein inhibitor, and SOCS proteins allow for multiple checkpoints and feedback inhibition [16]. However, loss of any counter-regulatory mechanisms can lead to uninhibited proliferation and survival. Constitutive STAT3 activation may confer resistance to apoptosis and promote cell cycle progression through its interactions with the IL-6 signal transducer, gp130 [73]. STAT3 activity is associated with upregulation of anti-apoptotic molecules such as Bcl-XL, Mcl-1, and survivin [52,74,75,76]. STAT3 inhibition has been shown to result in a concomitant reduction in the steady-state levels of anti-apoptotic molecules Bcl-XL, Bcl-2, and Mcl-1 [61]. Several *in vitro* and *in vivo* studies of STAT3 inhibitors suggest that STAT3 downregulation can induce cell cycle arrest and apoptosis, often in a dose-dependent manner [77,78,79]. Furthermore, STAT3 may be required for the maintenance of highly tumorigenic GBM stem cells (GBM-SC’s) [80]. Sherry *et al.* observed that even transient STAT3 inhibition results in irreversible growth arrest and loss of self-renewal capacities in GBM stem cells [81]. STAT3 tyrosine (Y) phosphorylation has also been associated with more aggressive tumors. Increased expression of Y705-phosphorylated STAT3 in GBM samples correlated with significantly shorter overall survival [82]. Together, there is consistent evidence that STAT3 is a key contributor to GBM pathogenesis by mediating cell survival, growth, and proliferation. 

### 4.2. Invasion

Infiltration and migration are characteristic of glioma tumor cell migration preferentially occurs along white matter tracts, accounting for the characteristic “butterfly lesions” frequently observed crossing the corpus callosum, perineuronal satellitosis, and perivascular or subpial spread [83]. STAT3 inhibitors have been shown to decrease GBM invasion in human glioma cell line U251 [74]. Using a JAK2 inhibitor and monolayer wound-healing assays, Senft *et al.* demonstrated decreased STAT3 activation and migratory behaviors across five different GBM cell lines *in vitro* [84]. Though the precise mechanisms remain unknown, STAT3 may contribute to invasion by upregulating pro-invasive factors such as matrix metalloproteinase-2 (MMP-2), MMP-9, and fascin-1 [42,84,85]. Increased STAT3 and focal adhesion kinase (FAK) has also been demonstrated in SOCS3 knockdown glioma cells leading to increased tumor invasion [40]. Further studies of the underlying signaling pathways are needed to more clearly define the relationship between STAT3 activation and GBM invasiveness.

### 4.3. Angiogenesis

VEGF-mediated angiogenesis is critical for the survival of most tumors, providing the requisite nutrients for accelerated growth and progression [24]. Constitutive tyrosine kinase activation is known to induce VEGF and has been observed in a wide range of cancers, including EGFR- and Src-induced VEGF in breast cancer [86,87], and IL-6 receptor associated kinases in myeloma [88]. Niu *et al.* noted that VEGF expression in several cancer cell lines correlated with constitutive STAT3 activity, and that STAT3 inhibition led to reduced Src-induced VEGF expression [89]. Results from promoter mutagenesis and chromatin immunoprecipitation assays further indicated that the VEGF gene is directly regulated by STAT3, thereby suggesting that STAT3-targeted therapy could play a significant role in disrupting tumor neovascularization. Studies of viral oncogenes have also alluded to the role of STAT3 activity in viral oncogene-mediated angiogenesis. Experiments with mouse fibroblast and human neural precursor cell lines have correlated human cytomegalovirus (HCMV)-induced VEGF expression to upregulation of STAT3, AKT, Erk1/2, FAK, Src, and endothelial nitric oxide synthase oncogenic pathways. More specifically, *in vitro* overexpression of the viral chemokine receptor US28 has been shown to activate several downstream transcription factors such as hypoxia inducible factor-1 and STAT3, resulting in VEGF promoter activation [90,91].

VEGF is often overexpressed in GBM [10,92,93], and pan-VEGF receptor tyrosine kinase inhibitors have been shown to normalize tumor vasculature and alleviate vasogenic brain edema [11]. Constitutively activated STAT3 and VEGF receptors can be coexpressed in glioma [58]. In a study of the phenotypic, physiologic, and molecular tumor response to bevacizumab, a recombinant humanized monoclonal anti-VEGF antibody, Keunen *et al.* found that vascular remodeling and the creation of a more hypoxic tumor microenvironment paradoxically resulted in a more invasive, glycolysis-dependent tumor phenotype [93]. De Groot *et al.* demonstrated that anti-VEGF therapy in glioma patients results in markedly elevated levels of STAT3 expression, and that STAT3 inhibitors could enhance the efficacy of antiangiogenic treatment strategies [94]. Further study of the VEGF and STAT3 interactions will be required to better understand tumor escape and resistance patterns.

### 4.4. Immune Suppression and Evasion

Tumor-mediated immune suppression at both the local and systemic levels has been well described in GBM patients [17]. T cell anergy, lymphodepletion, lymphosuppression, and impaired antibody synthesis are just some of the ways in which GBMs avoid antigen recognition and targeted destruction [39].

STAT3 has been established as an important negative regulator of the host’s antitumor immune response. STAT3-associated immunosuppressive mediators generally fall into two categories: (1) soluble or membrane-bound proteins produced by glioma cells and (2) immune cell populations recruited by the tumor. Secreted factors include IL-10 (an inhibitor of Th1 activity), VEGF, and TGF-beta, which inhibit T cell, B cell, natural killer (NK) cell, and monocyte functions [49,95,96]. VEGF not only promotes angiogenesis, as described previously, but also establishes a positive feedback for enhanced STAT3 activation in immature dendritic cells (DCs) [97]. STAT3 hyperactivity can lead to abnormal DC differentiation via constitutive JAK2/STAT3 activation [18] and decreased expression of major histocompatibility complex (MHC) class II, costimulatory CD40, and IL-12 molecules [98]. Conversely, STAT3 blockade in human GBM cell lines has been shown to alter pro-inflammatory cytokine and immune cell profiles. A study by See *et al.* demonstrated that siRNA-mediated STAT3 suppression triggered the release of soluble factors such as interferon (IFN) gamma-inducible protein 10 (IP-10), Rantes, IL-8, IL-6, tumor necrosis factor (TNF)-α and interferon (IFN)-β; furthermore, exposure to these paracrine signaling molecules induced DC maturation and activation [17].

Microglia (MG) make up almost one third of the non-tumor cells found in GBM tissue sections and are responsible for secreting MMP-9, EGF, and VEGF. In the homeostatic state, MG act as resident antigen presenting cells with proinflammatory, anti-neoplastic functions. However, relatively recent evidence suggests that exposure to glioma-derived chemokines induce constitutive STAT3 activation in MG, with consequent suppression of antitumor mechanisms or even tolerance to tumor antigens [99,100,101]. In contrast, a study by Komohara *et al.* demonstrated that direct interaction with glioma cells led to STAT3 activation in tumor infiltrating macrophages. In return, MG/macrophage-derived factors were shown to activate STAT3 signals in tumor cells, perpetuating glioma pathogenesis and progression [102].

STAT3 also recruits and promotes the proliferation of T regulatory cells (Tregs), which suppress effector lymphocyte activity within the tumor microenvironment [103,104]. Tregs are preferentially recruited to high-grade gliomas following tumor secretion of CCL2/22 chemokines [105]. Following direct interaction with local dendritic cells, Tregs secrete TGFb to inhibit activation of cytotoxic T cells [19,106]. STAT3 deletion in hematopoietic cells has been associated with a markedly decreased number of tumor-infiltrating Treg cells, as well as enhanced activity of DCs, natural killer (NK) cells, T cells, and neutrophils [107]. These findings further implicate STAT3 as a negative regulator of the host immune system.

## 5. STAT3 as a Tumor Suppressor

While STAT3 activation is clearly implicated in GBM survival through invasion, differentiation, angiogenesis, and immunosuppression, recent studies in GBM and other tumors have suggested a role for STAT3 in tumor suppression. Most studies illustrate STAT3 involvement in growth inhibitory signaling, terminal cell differentiation, and apoptosis. For example, the IL-6 growth-inhibitory and terminal differentiation signal has been correlated with STAT3 transcriptional activity in prostate cancer [108,109]. Similarly, IL-6 mediated growth arrest in melanoma is STAT3-dependent [110]. STAT3 is also involved in myeloid cell differentiation via granulocyte colony stimulating factor (G-CSF)-mediated JAK phosphorylation and p27 upregulation [111,112,113].

In stratified squamous epithelium, constitutively active STAT3 is responsible for terminal differentiation through enhanced activation of keratin 13. Inhibition of STAT3 via overexpression of the tumor suppressor PTEN promotes proliferation and tumorigenesis [114]. Moreover, STAT3 activation is implicated in epithelial cell apoptosis in the mammary gland, and STAT3 knockout in mammary gland tissue results in delayed involution suggesting an early role for STAT3 in initiation of apoptosis [115]. Keratinocyte differentiation involves STAT3 activation and its association with p27kip1 accumulation, while hepatocyte epithelial tubule differentiation relies on hepatocyte growth factor-mediated STAT3 activation and translocation to the nucleus [116,117]. GBM is not precluded from such STAT3-mediated cell cycle regulation, and recent studies suggest that STAT3 activation actually prevents malignant transformation of glial cells in some systems [118,119].

The role of the JAK-STAT pathway is well established in astrocyte differentiation [120,121,122,123,124]. While STAT3 activation has been associated with GBM evasion of immunosurveillance and enhancement of cell survival and proliferation, it is not surprising that STAT3 activation suppresses malignant transformation in certain GBM genotypes. De la Iglesia *et al.* have shown that in PTEN-deficient GBM, endogenous STAT3 inhibition prevents STAT3-mediated transcriptional IL-8 repression, resulting in increased tumor proliferation and invasiveness [118]. Furthermore, they found that reactivation of STAT3 in PTEN-deficient GBM suppresses the invasive phenotype and GBM proliferation along myelin in white matter tracts. Thus, whether STAT3 behaves as a tumor suppressor or oncogene relies on the tumor genetic background—activation of STAT3 in PTEN deficient GBM may actually prevent tumor invasion and stabilize tumor growth.

## 6. STAT3 as a Prognostic Indicator

Given STAT3’s established role in GBM tumorigenesis and tumor suppression, it is a reasonable candidate as a prognostic marker. Jin *et al.* report that STAT3 expression in colorectal carcinoma is correlated with higher-grade tumors and poorer survival [125]. Conversely, Gordziel and colleagues found that strong STAT3 expression in colorectal carcinoma biopsies is associated with an improvement in median survival of about 30 months compared to STAT3 negative biopsies [126]. Such discrepancies in study results may reflect the dependence of STAT3 tumorigenic *versus* tumor suppressor function on the tumor genetic background, potentially confounding the results.

In GBM, survivin positivity has been suggested as a strong prognostic indicator of significantly poorer survival and higher malignant grade, owing to its anti-apoptotic activity [127,128]. Regarding STAT3, Abou-Ghazal and colleagues found that pSTAT3 expression in astrocytomas was correlated with poorer survival, and Tu *et al.* report that JAK/STAT activation correlates with higher-grade gliomas and is an independent prognostic indicator of decreased survival [56,129]. Recent studies demonstrate that STAT3 could be a useful tumor marker of poor prognosis; however, given the tumor suppressive role of STAT3 in PTEN deficient tumors, it is important that future studies stratify GBM samples based on genetic background, as the relevance of STAT3 to tumorigenesis is a function of GBM genotype.

## 7. STAT3 as a Therapeutic Target

A number of approaches have been developed to target STAT3 or its downstream effects and thereby inhibit glioma growth. Specific antitumor therapies against STAT3 are summarized in Table 2.

**Table 2:** Anti-tumor therapies targeting STAT3.

Drug	Mechanism of Action
Oleanolic acid	Suppresses IL-10 secretion which suppresses M2 polarization of tumor-associated macrophages [130]
LLL12	Suppress phosphorylation of STAT3; inhibit STAT3 DNA binding [26,131]
LLL3	
WP1193	Inhibitor of JAK2/STAT3 pathway in glioma-like stem cells resultin gin G1 arrest [132]
RNAi	Downregulation of cyclin D1 in glioma cells [133]
Oligodeoxynucleotides	Induce cell cycle arrest and apoptosis by mimicking STAT3 specific cis elements [36]
AG490	Inhibits JAK2, resulting in decreased activation of STAT3 and downstream decreased expression of MMP-2 and MMP-9 and inhibition of tumor cell invasiveness [84]

Potential drugs that directly inhibit STAT3 activation include the naturally occurring triterpenoid oleanolic acid, which also suppresses the M2 polarization of tumor-associated macrophages by suppressing IL-10 secretion [130]. Small molecule inhibitors are also candidates for suppressing STAT3 activity, as they produce a similar anti-tumor inflammatory microenvironment as siRNA-mediated knockdown of STAT3 [17]. Other small molecule inhibitors, including LLL12 and LLL3, suppress phosphorylation of STAT3 and inhibit STAT3 DNA binding, resulting in decreased viability of tumor cells and resultant apoptosis. Moreover, treatment with LLL3 increased survival in GBM-bearing mice by 12.5 days [26,131]. Others have targeted the JAK2/STAT3 pathway in glioma stem-like cells (GSCs). WP1193 is a small molecule inhibitor of JAK2/STAT3, which promotes *in vivo* glioma inhibition in a dose-dependent manner and is partially associated with G1 arrest in GSCs [132]. STAT3 knockdown with interfering RNA, delivered by a lentivirus vector, resulted in down-regulation of cyclin D1 and inhibition of glioma cell proliferation [133]. Oligodeoxynucleotides may also serve as future therapeutic options as they induce cell-cycle arrest and apoptosis by mimicking STAT3 specific cis-elements [36]. Furthermore, STAT3’s role in Th17 T cell differentiation and cytokine production renders it an attractive target for immunotherapy in autoimmune pathways, as ablation of STAT3 in CD4 cells results in increased Th1 responses rather than Th17 responses [134].

Other drug targets include STAT3 associated genes that inhibit tumor cell migration or invasion [135]. AG490 inhibits JAK2, the upstream activator of STAT3, which results in decreased expression of the STAT3 regulated genes MMP-2 and MMP-9 [84].

STAT3 inhibition is also useful synergistically with other treatment modalities. Resveratrol, a grape polyphenol, has been shown to enhance glioma radiosensitivity by inhibiting STAT3 signaling, rendering future promise for more effective radiotherapy [136]. Resistance to temozolomide has been shown to be associated with STAT3 activation and upregulation of the DNA repair enzyme MGMT [14]. Thus, STAT3 knockdown prior to temozolomide therapy may reduce the incidence of tumor resistance to chemotherapy.

While STAT3 is clearly an integral pathway to tumor growth and invasion, lifting the “brakes” on immune function through STAT3 checkpoint blockade combined with tumor-specific vaccine therapy may show promise for more robust anti-tumor responses [16]. Such checkpoint blockade combined with vaccine based immune activation has shown enhanced anti-tumor responses over vaccine therapy alone in mouse models and clinical trials combining cytotoxic-T-lymphocyte-associated protein 4 (CTLA-4) blockade and granulocyte/macrophage-colony stimulating factor-secreting tumor vaccines [16,134,137,138,139,140,141]. Combination blockade of STAT3 along with vaccine therapy has shown encouraging results in melanoma [134]. Thus, multi-modality therapy involving cancer vaccines may be the key to the role of STAT3 inhibition in curative immunotherapy by promoting CD4 and CD8 T cell mediated tumor-specific killing [140].

## 8. Conclusions

Recent studies continue to define the role of STAT3 as a constitutively active element in gliomagenesis. Activation of STAT3 has been implicated in tumor activation through the production of anti-apoptotic and glioma stem-cell maintenance factors, pro-invasive enzymes, and angiogenic elements such as VEGF. Additionally, STAT3 helps to orchestrate immune evasion of GBM through downstream increases in Tregs and decreases in activated circulating lymphocytes. On the other hand, studies have also shown that STAT3 may paradoxically facilitate tumor suppression by contributing to cell differentiation, growth inhibitory signaling, and apoptosis. It is important to note that much of the evidence supporting STAT3’s role as a potential tumor suppressor has been obtained through *in vitro* cell line experiments, which may limit its relevance for clinical GBM pathogenesis. Further research on the pro- and anti-tumorigenic effects of its activation is needed to maximize the possibilities of STAT3 as a prognostic indicator and a target for molecular therapy.
